# Ten tips to reduce fracture risk by non-pharmacologic interventions in CKD-associated osteoporosis

**DOI:** 10.1093/ckj/sfag303

**Published:** 2026-09-01

**Authors:** Ditte Hansen, Hanne Skou-Jørgensen, Mehmet Kanbay, Maria Fusaro, Louise Salomo, Susanne Fleig, Stig Molsted, Jesper Ryg, Mathias Haarhaus, Pieter Evenepoel

**Affiliations:** Department of Nephrology, Copenhagen University Hospital – Herlev, Copenhagen, Denmark; Institute of Clinical Medicine, University of Copenhagen, Copenhagen, Denmark; Department of Clinical Medicine, Aarhus University, Aarhus, Denmark; Department of Renal Medicine, Aarhus University Hospital, Aarhus, Denmark; Department of Clinical Medicine, Aalborg University, Aalborg, Denmark; Department of Internal Medicine, Division of Nephrology, Koc University School of Medicine, Istanbul, Turkey; National Research Council (CNR), Institute of Clinical Physiology (IFC), Pisa, Italy; Department of Medicine, University of Padova, Padova, Italy; Department of Nephrology, Copenhagen University Hospital – Herlev, Copenhagen, Denmark; Department of Nephrology and Clinical Immunology, RWTH Aachen University Hospital, Aachen, Germany; Institute of Clinical Medicine, University of Copenhagen, Copenhagen, Denmark; Department of Physiotherapy and Occupational Therapy, Copenhagen University Hospital – North Zealand, Denmark; Institute of Clinical Medicine, University of Copenhagen, Copenhagen, Denmark; Geriatric Research and Clinical Evidence (GRACE), Department of Internal Medicine Geriatric Section, Copenhagen University Hospital – Herlev and Gentofte Hospital, Copenhagen, Denmark; Geriatric Research Unit, Department of Clinical Research, University of Southern Denmark, Odense, Denmark; Division of Renal Medicine, Department of Clinical Science, Intervention and Technology, Karolinska Institutet, Karolinska Universitetssjukhuset, Stockholm, Sweden; Tiohundra AB, Norrtälje, Sweden; Department of Microbiology, Immunology and Transplantation, Nephrology and Renal Transplantation Research Group, Katholieke Universiteit Leuven, Leuven, Belgium; Department of Medicine, Division of Nephrology, University Hospitals Leuven, Leuven, Belgium

**Keywords:** calcium, CKD-MBD, chronic renal failure, mineral metabolism, vitamin D

## Abstract

Chronic kidney disease (CKD)-associated osteoporosis is present in approximately 25% of all patients with CKD. The occurrence increases as kidney function declines, with prevalences up to 75% in elderly dialysis patients. It is often undiagnosed due to the perception that limited treatment options are available for this condition. This inappropriate assumption leads to diagnostic and therapeutic nihilism. The effect-size of pharmacological interventions on bone mineral density in patients with CKD is comparable to or higher than in other forms of osteoporosis. Non-pharmacological interventions can improve bone strength and fracture risk in addition to pharmacological interventions. Importantly, they are often applicable to patients with CKD without restrictions, with some specific considerations in end-stage kidney disease. Due to the increased prevalence of modifiable risk factors for fragility fractures in CKD, non-pharmacological interventions should be provided to all patients with CKD-associated osteoporosis. We review the current literature and describe 10 tips for non-pharmacological interventions with the potential to modify fracture risk in CKD patients: appropriate intake of calcium and vitamin D, adequate nutrition and protein intake, avoidance of smoking and alcohol, medication review, reinforcement of exercise, reducing risk of home hazards, prevention of orthostatic hypotension, taking care of neuropathy and determining fall risk, as well as implementing fracture liaison services, are all actions of relevance to the patient with CKD-associated osteoporosis. As in the general population, non-pharmacological interventions should be the first option to consider when osteoporosis is diagnosed. With this paper, we suggest how this should be tailored to the patient with CKD.

## Graphical Abstract

**Graphical Abstract:**
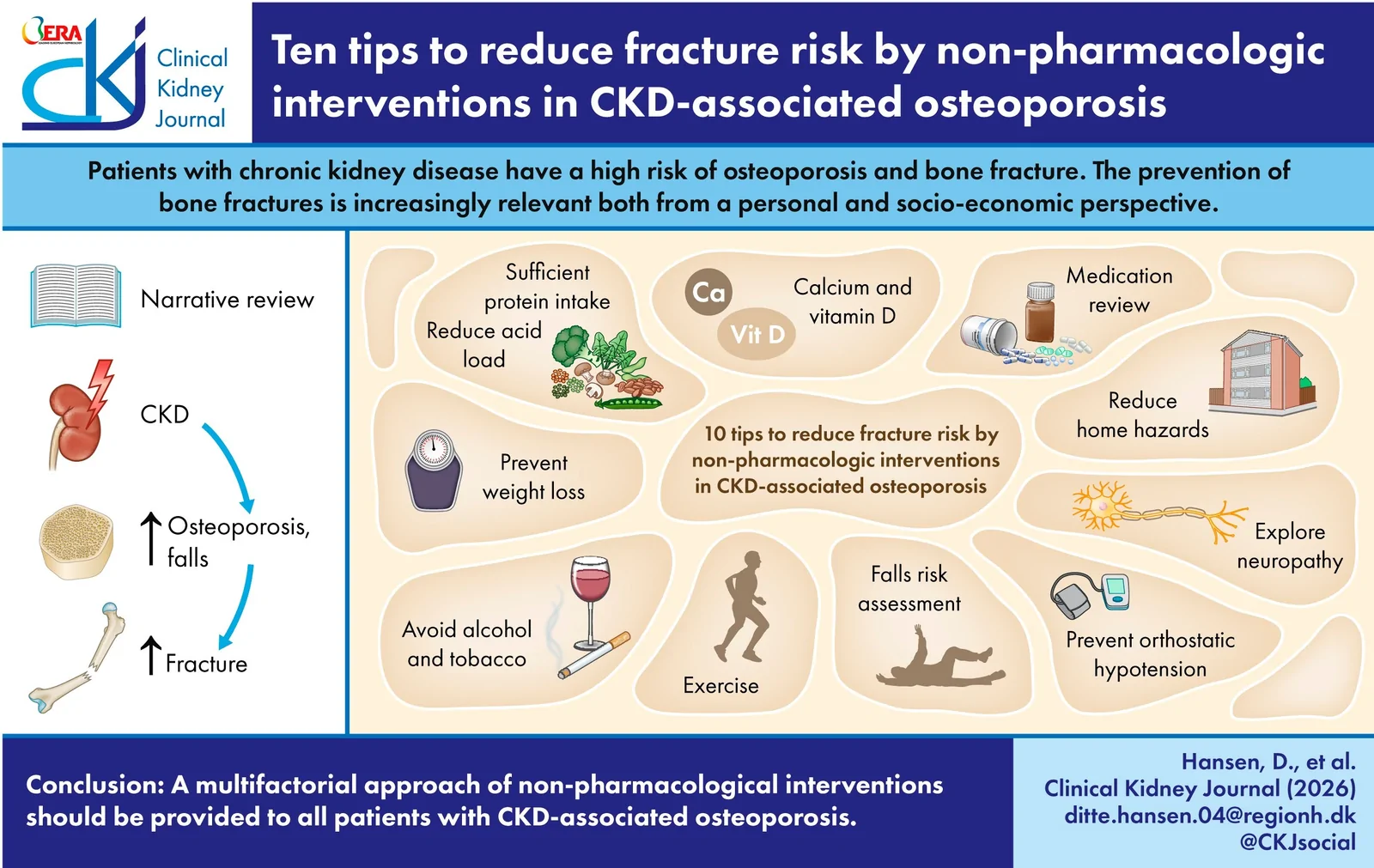

## INTRODUCTION

The risk of fracture increases as kidney function declines and is particularly high in kidney failure, with patients receiving dialysis having a 3-fold increased fracture risk compared with the general population [1, 2]. The prevalence of chronic kidney disease (CKD), osteoporosis, falls and fractures all increase with age, and as the population grows older, the prevention of bone fracture becomes increasingly relevant both from a personal and a socio-economic perspective.

CKD-associated osteoporosis is a distinct entity of reduced bone strength resulting in increased fracture risk in the setting of kidney dysfunction, which integrates the impact of both traditional and CKD-specific risk factors [3]. While the current paradigm promotes a more liberal use of bone-targeting drugs [3], non-pharmacological strategies remain the first-line approach for all patients with osteoporosis.

While non-pharmacological strategies to a large extent are interchangeable between different subtypes of osteoporosis, the presence of CKD G3b–5D warrants some modifications related to specificities of the condition, including dietary restrictions, disturbances in mineral metabolism, polypharmacy and dialysis. The aim of this paper is to provide an overview of 10 non-pharmacologic interventions to reduce the fracture risks in patients with CKD (Fig. 1 and Table 1).

**Figure 1:**
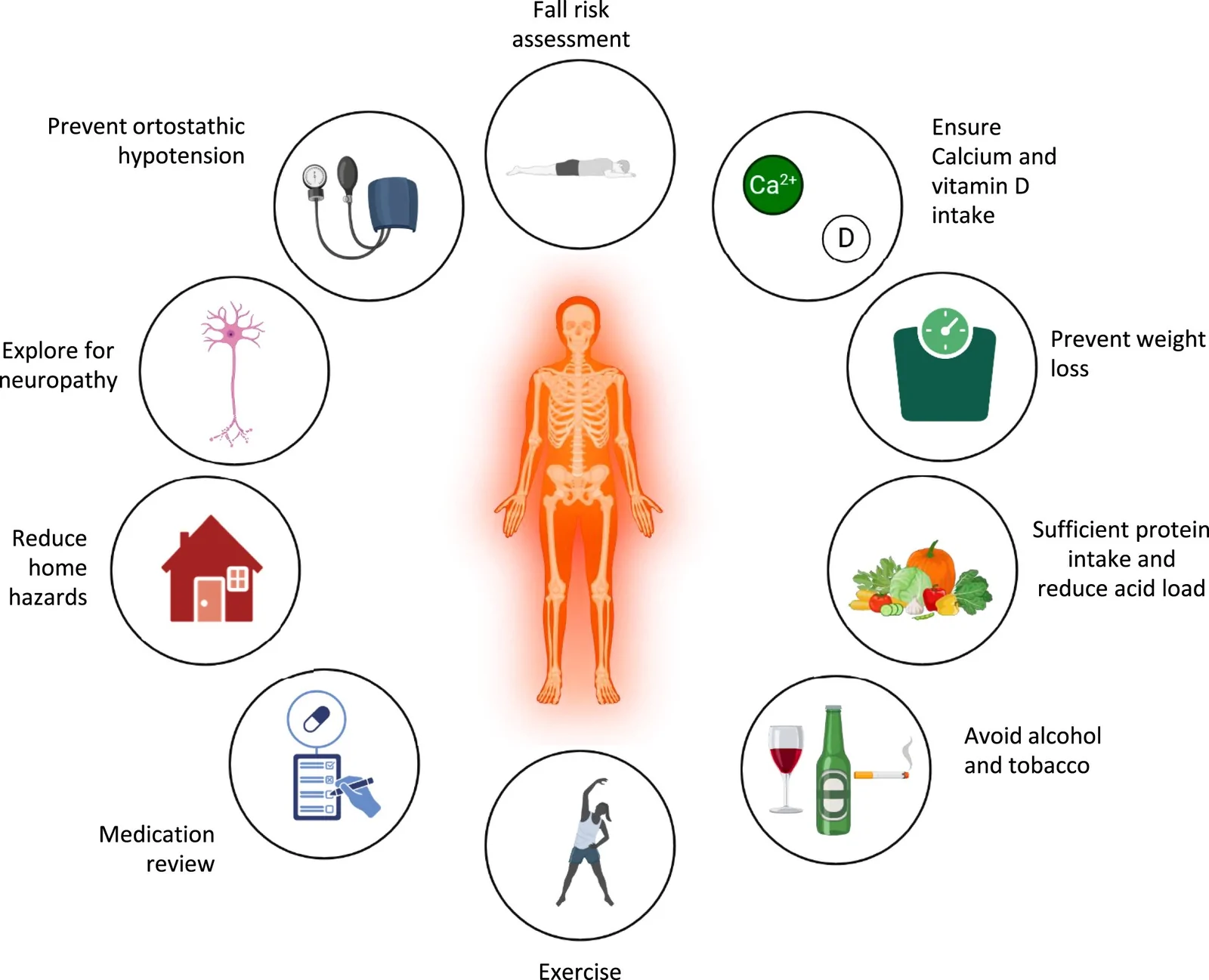
Ten tips for non-pharmacological interventions in patients with CKD-associated osteoporosis.

**Table 1:** Summary of evidence for non-pharmacological interventions in CKD-associated osteoporosis.

			Effect on surrogate outcome	Effect on fracture		
Tip	Intervention	CKD grade	CKD	General population	CKD	Cautions
1	Calcium intake	1–5D	↓ Hyperparathyroidism (RCT), Essential for growth (NRSI)	↓ When intake is low (RCT)	UN	High calcium intake (>1500 mg/day) may confer risk of hypercalcaemia and vascular calcification
	Vitamin D supplementation	1–5D	↓ Hyperparathyroidism (RCT), essential for bone mineralisation (RCT), ↑ BMD (RCT)	No effect when 25OH vitamin >50 nmol/L (RCT)	↓ in KTX (RCT)	
2	Sufficient nutrition	4–5D	↓ Sarcopaenia and falls (NRSI)	↓ Nursing home (RCT)	UN	
3	Reduce acid load	3b-5	↑ BMD (NRSI), ↓ falls (NRSI)	UN	↓ (NRSI)	
	Adequate protein intake	1–5D	Low protein intake assoc. w sarcopaenia, no effect on BMD (RCT)	↓ (NRSI) and nursing home (RCT)	UN	High protein intake in CKDnD may increase progression of CKD
4	Avoid smoking	1–5D	Smoking assoc. w ↓ BMD and calcium absorption (NRSI)	↓ (NRSI)	↓ (NRSI)	
	Avoid alcohol	1–5D	↓ Falls, ↔ BMD (NRSI)	↓ (NRSI)	UN	
5	Advice exercise	1–5D	↑ BMD (NRSI)	↓ (RCT)	UN	
6	Medication review and deprescribing	1–5D	UN	UN	Polypharmacy assoc. w fracture (NRSI) and withdrawal assoc. w ↓ fracture	Drug efficacy weighted against safety
7	Fall prevention	1–5D	UN, reduce falls in non-CKD (RCT)	UN	UN	
8	Explore for neuropathy	5D	UN, assoc. w falls in non-CKD (NSRI)	UN	UN	
9	Ortostatic hypotension	1–5D	UN, assoc. w falls in non-CKD (NSRI)	↑ (NRSI)	UN	
10	Fall risk and FLS	1–5D	UN	↓ (NRSI)	UN	

RCT, randomized controlled trial; NSRI, non-randomized controlled studies: UN, unknown; BMD, bone mineral density; KTX, kidney transplanted patient; assoc. w, associated with; CKDnD, CKD not on dialysis.

## SUMMARY OF 10 TIPS TO PREVENT FRACTURES BY NON-PHARMACOLOGICAL INTERVENTIONS IN PATIENTS WITH CHRONIC KIDNEY DISEASE

### Tip 1: Ensure sufficient calcium and vitamin D intake

A sufficient intake of calcium and vitamin D are considered crucial for maintaining skeletal health (Fig. 2), and supplements are routinely used both for primary prevention and treatment as add-on to bone-specific medical drugs of post-menopausal and other forms of osteoporosis [4]. In CKD, calcium homeostasis is dysregulated, and both a positive and a negative calcium balance carry risk. Calcium loading has been shown to drive cardiovascular calcification in patients with CKD [5–8] and should be avoided. On the other hand, calcium deficiency can drive secondary hyperparathyroidism [9, 10] and potentially contribute to skeletal fragility. A recent European consensus paper provided a thorough review of current literature and concluded that a daily calcium intake of 800–1000 mg, not exceeding 1500 mg, is recommended across all grades of CKD [9]. The average dietary calcium intake in adults with CKD G4–5D is estimated at 400–800 mg/day [11] and below the recommendation [12]. However, total calcium intake increases substantially with the use of calcium-containing phosphate binders [13–15]. Thus, dietary intake, supplements and other calcium-containing medications must be considered for total calcium intake. A quick estimation of calcium intake can be gained from questionnaires [16] or online calculators [17, 18], adapted to local needs in the specific country. Importantly, young adults (until mid-20s) may need a greater supply of calcium due to the needs of their growing skeleton [19, 20].

**Figure 2:**
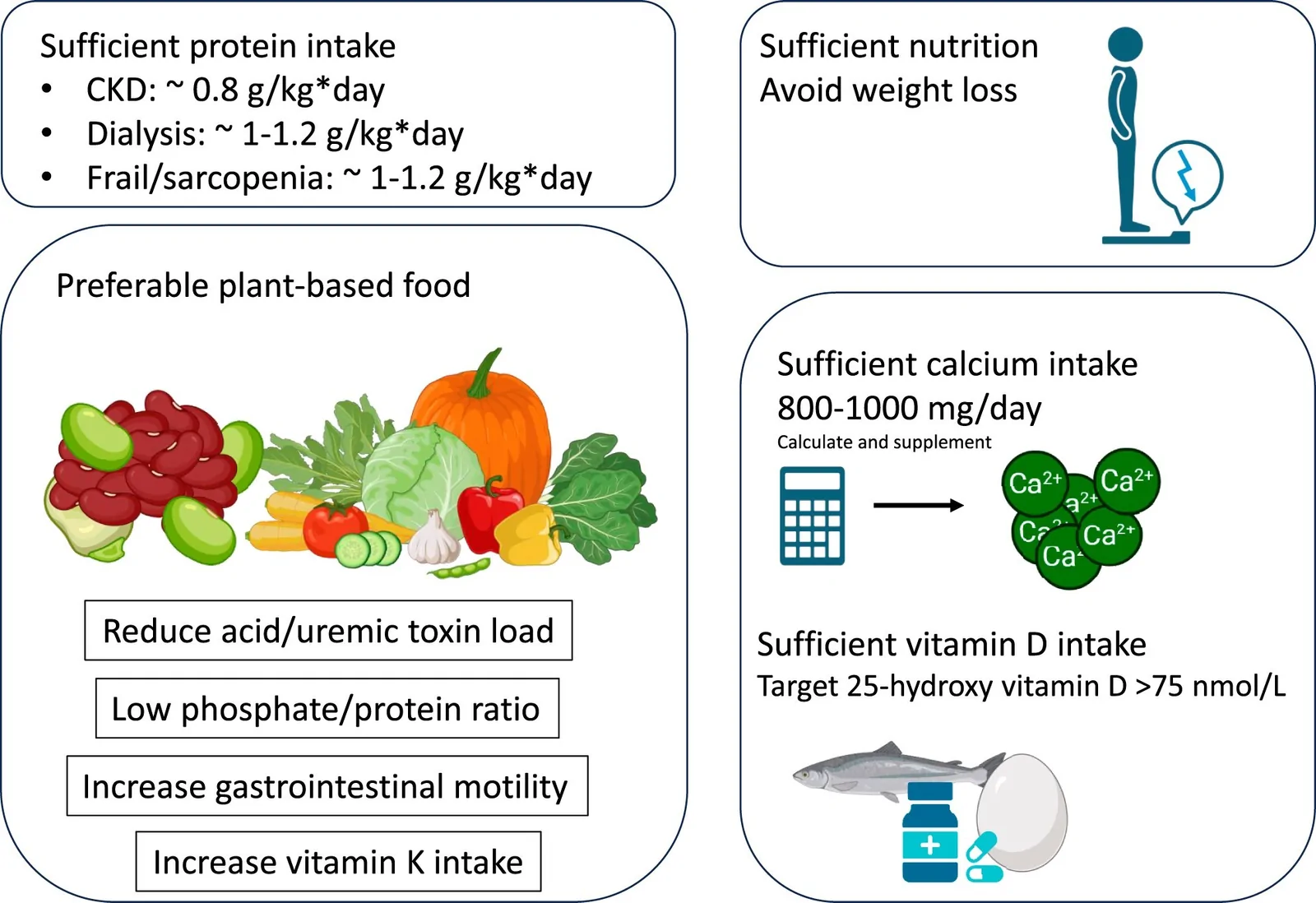
The healthy bone diet in chronic kidney disease. Summary of dietary interventions to protect the bone in patients with CKD (25-hydroxy vitamin D >75 nmol/L equals >30 ng/mL).

Active vitamin D is needed for efficient uptake of calcium from the gastrointestinal tract. Thus, insufficient vitamin D levels stimulate parathyroid hormone release to enable calcium mobilization from the bones [21]. Vitamin D is also necessary for normal bone mineralization, and severe vitamin D deficiency results in the clinical syndromes of rickets in children and osteomalacia in adults [22]. In CKD, maintaining sufficient vitamin D levels improves the control of secondary hyperparathyroidism [23–25] and has been shown to reduce the risk of bone loss [24] and fractures [26] after kidney transplantation. Even with the reductions in functional kidney mass, patients with CKD can utilize nutritional vitamin D, as demonstrated by the presence of relevant levels of active vitamin D even in patients who are anephric [27], and increases in these levels when nutritional supplements are given to patients receiving dialysis [28]. A recent European consensus paper recommended providing nutritional vitamin D to a target level of 25-hydroxy vitamin D >75 nmol/L (30 ng/mL) across all grades of CKD [29]. Increased intake of fatty fish and eggs may increase the level of 25-hydroxy vitamin D, but supplements will often be necessary. Excessive sun exposure is usually not recommended due to the increased risk of skin cancer, especially in relation to immunosuppressants and hydrochlorothiazide.

Due to several disease-specific conditions, CKD patients typically exhibit lower vitamin K (K1 and K2) levels than the general population. This deficiency is associated with a higher prevalence of bone fractures, vascular calcifications and increased mortality. An adequate intake of vitamin K could potentially mitigate CKD–mineral and bone disorder in CKD patients [30]. Vitamin K occurs mostly in green vegetables, fruits and plant oil, but the dietary intake may not be sufficient to supplement vitamin K deficiency [31].

### Tip 2: Ensure sufficient nutrition to prevent weight loss

Preservation of nutritional status is a cornerstone of bone health (Fig. 2). Unintentional weight loss, low body mass index and protein-energy wasting are highly prevalent conditions in CKD G4–5D and strongly associated with frailty, sarcopaenia, falls, fractures, hospitalization and mortality [32]. Beyond muscle loss, inadequate energy intake compromises the bone–muscle unit, reducing mechanical loading and accelerating skeletal fragility [33]. Prevention of weight loss must therefore be one of the primary treatment goals.

Routine monitoring of body weight (BW) and body mass index with trends over time is recommended. Unintended weight loss of >5% over 3–6 months should trigger attention. Appetite loss, early satiety, inflammation, intercurrent illness and dialysis-related catabolism are common contributors to weight loss, which can be actively addressed.

Energy intake should generally be maintained at approximately 30–35 kcal/kg/day in non-obese adults, with individual adjustment according to age, physical activity and comorbidity [34, 35]. In patients with reduced appetite, practical strategies include small frequent meals, energy-dense foods, oral nutritional supplements when needed and avoidance of unnecessary dietary restrictions. Dietary phosphorus intake may also influence bone. Food items with lower phosphorus-to-protein ratio should be preferred to control hyperparathyroidism while not compromising protein intake [36].

Dietary recommendations in advanced CKD frequently require balancing competing priorities, including adequate protein and energy intake, phosphate control, potassium management, glycaemic control and treatment of protein-energy wasting. Therefore, nutritional advice should always be individualized according to CKD stage, dialysis status, nutritional status and biochemical parameters (particularly serum potassium, phosphate and bicarbonate). Early referral to a renal dietitian is recommended, especially in patients receiving dialysis therapy, to individualize dietary prescriptions and secure sufficient nutrition.

In patients with CKD G4–5D, preventing unintended weight loss may support overall musculoskeletal health by helping maintain energy stores and muscle mass.

### Tip 3: Ensure low acid load and sufficient protein intake

In CKD G3b–5, impaired renal acid excretion and reduced bicarbonate generation lead to acid retention and metabolic acidosis. Bone buffers excess acid, causing mineral loss and reduced bone quality [37]. Acidosis also accelerates muscle wasting by increasing protein breakdown and inhibiting synthesis [38]. Metabolic acidosis is associated with increased risk of falls and fracture in CKD [39], therefore preventing acidosis may be an important fracture risk–lowering strategy in CKD (Fig. 2).

Although renal acid excretion cannot be increased, diet strongly influences net endogenous acid load. Plant-based foods, especially vegetables and fruit, provide alkaline load and fibre; regular intake is linked to an 8% reduction in hip fracture risk [40, 41]. In CKD G3b–G4, high fruit and vegetable intake (sufficient to halve dietary acid load) corrected metabolic acidosis as effectively as bicarbonate [42]. However, in patients with CKD G4–5D or hyperkalaemia, increasing fruit and vegetable intake should be individualized according to serum potassium levels and, where appropriate, focus on lower-potassium choices and preparation methods that reduce potassium content. If hyperkalaemia persists despite individualized dietary counselling, potassium binders may be considered to facilitate a healthy but less restrictive diet.

Plant protein sources generate lower acid load and contain less phosphorus with lower bioavailability than animal protein [43]. The main dietary acid sources are meat, eggs and cheese. Shifting protein intake toward plant sources reduces acid load, while at the same time reducing uraemic toxin production via favourable effects on the gut microbiota. Dietary fibre fermentation to short-chain fatty acids supports colonic epithelial energy needs and strengthens gut barrier integrity [41]. An example of a diet integrating these principles is the new Nordic renal diet [44].

Guidelines recommend a protein intake of 0.8 g/kg BW/day for most patients with CKD not on dialysis [45], the same amount as recommended by the World Health Organization to preserve muscle mass in healthy adults [46]. While dietary protein restriction has traditionally been recommended to slow CKD progression, the supporting evidence remains modest, except for very low-protein diets supplemented with ketoanalogue amino acids in selected patients [47, 48]. However, implementation of very low-protein diets requires close follow-up by an experienced renal dietitian to ensure nutritional adequacy and adherence, and ketoanalogue supplements are not universally available, limiting the feasibility of this approach in routine clinical practice. Recent observational evidence suggests that, in older adults with mild-to-moderate CKD and in patients receiving dialysis therapy, or when malnutrition or sarcopaenia are present, higher protein amounts are recommended: 1–1.2 g/kg BW/day in dialysis, going up to 1.5 g/kg BW/day or higher if the aim is to treat malnutrition and sarcopaenia [49]. Although a direct effect of protein intake on bone density has not been demonstrated [50], the prevention of malnutrition and sarcopaenia may reduce the risk of falls and fracture.

### Tip 4: Reduce smoking and alcohol

Smoking is a well-established risk factor for numerous health problems, including osteoporosis and fracture [51]. Although tobacco smoke contains more than 4000 chemical compounds, the specific constituents responsible for skeletal harm when smoking remain poorly defined. Smoking affects bone mass indirectly by altering BW, disrupting the parathyroid hormone–vitamin D axis, and modifying adrenal and sex hormone levels, while also increasing oxidative stress. In addition, tobacco smoke may exert direct deleterious effects on bone cells, including a negative effect on osteoblasts, through nicotinic and aryl hydrocarbon receptor signalling [52, 53].

Moderate alcohol consumption has not been shown to adversely affect bone health and may even be associated with slightly higher bone density and reduced fracture risk in post-menopausal women. In contrast, higher levels of alcohol intake—more than two drinks per day for women or three for men—can be detrimental. Excessive alcohol use has been linked to impaired calcium absorption, increased fall risk, and secondary factors such as malnutrition, reduced physical activity and hormonal disturbances, all of which contribute to elevated fracture risk [54].

Although it remains unclear whether CKD amplifies the skeletal effects of smoking or alcohol consumption, avoidance of smoking and alcohol consumption should nonetheless be advised for individuals with CKD, just as in the general population. These measures are important not only for the prevention and management of (CKD-associated) osteoporosis and fractures [55], but also for reducing the broader spectrum of health risks linked to these behaviours.

### Tip 5: Advise exercise training

Exercise reduces the rate of falls and fractures in the general population [56]. There is robust evidence that exercise training improves aerobic capacity, quality of life and walking ability across CKD grades [57]. These benefits extend to patients receiving dialysis, who can engage in training either during or outside dialysis sessions [58].

In patients with CKD G3–5 (including those on dialysis and after kidney transplantation) there is a positive association between physical activity level and bone mineral density as well as beneficial effects of exercise training on bone biomarkers [59]. Resistance training improves bone mineral density in post-menopausal women, whereas combined exercise categories improve spine bone mineral density [60], we expect the similar pattern in CKD, even though this has not been sufficiently investigated.

A randomized trial in patients treated with haemodialysis, performing thrice-weekly exercise for 3 years, fractures were reported in 10% of controls versus none in the exercise group; however, the result was not statistically significant [61].

Exercise may also reduce risk of falls [62]. A randomized trial in patients with CKD G4 demonstrated improved balance with exercise [63]. Exercise improves physical function, muscle strength and improve muscle contraction, all parameters that are expected to reduce the risk of falls [64–66]. Lower-extremity coordination exercises with ‘gamification’ (integration of game elements like points, badges, leaderboards and challenges into non-game context) to enhance motivation for training during dialysis is a new approach, which have been shown to improve postural sway in older individuals [67].

Further research is needed to clarify the direct impact of exercise training on fracture risk in CKD. However, the observed benefits on bone health, falls and balance suggest a strong link to fracture prevention. Therefore, exercise training should be recommended for patients with CKD across all grades, as it may not only reduce the fracture risk but also improve other important health outcomes. For patients not having their own initiative to engage in training, organized exercise programs and sessions should be available. Training during or in relation to dialysis sessions is an efficient way to increase the amount of exercise for the patient on dialysis.

### Tip 6: Conduct regular medication review and deprescribing

Polypharmacy is common in CKD, with a prevalence of 69% in patients with CKD G1–5D [68] and a median daily pill burden of 19 in those receiving dialysis [69]. Polypharmacy is associated with frailty and an increased risk of falls and fracture in the older adult population [70, 71]. Similarly, in patients with CKD, a higher number of medications is associated with a greater risk of falls and fractures [70, 72]. Some of the frequently used medications in patients with CKD are known to negatively affect bone health (Table 2). In particular, glucocorticoids remain a cornerstone therapy in autoimmune conditions such as glomerulonephritis and following kidney transplantation. Higher cumulative glucocorticoid exposure associates with an increased fracture risk, whereas early withdrawal after kidney transplantation associates with reduced risk [73]. Use of proton pump inhibitors associates with an increased fracture risk in patients receiving dialysis therapy [2], potentially due to reduced gastrointestinal absorption of calcium and magnesium, or increased risk of falls, mediated by neuropathy due to B12 deficiency. Whereas loop diuretics increase renal calcium excretion and associate with an increased risk of fracture in the general population, this association was not found in a recent Dialysis Outcomes and Practice Patterns Study (DOPPS) study, which is in line with the negligible urinary calcium excretion [74].

**Table 2:** Drugs that may increase risk of (1) falls and (2) bone fragility in patients with CKD including examples of associated mechanisms [75, 116].

Drug	Mechanism	CKD specific consideration
Alpha-blockers	1. Hypotension, dizziness, blurred vision, sleep disorders, arrhythmia	Often used as antihypertensives
Anticholinergics	1. Sedation, confusion, dizziness, cognitive impairment, impaired concentration, blurred vision, tachycardia	
Antidepressants	1. Drowsiness, fatigue, dizziness, imbalance, diplopia, cognitive impairment, confusion, drug-induced movement disorders, hyponatraemia	
	2. Accelerated BMD decline	
Androgen-deprivation therapy	2. Hypogonadism leading to high bone turnover	
Antihistamines	1. Sedation, vertigo, orthostatic hypotension, hypotension, drug-induced movement disorders, cardiac effects (QTc prolongation and tachycardia), anticholinergic effects, hyponatraemia	
Anticoagulants	2. Heparin: reduced bone formation and increased bone resorption; warfarin: impaired mineralization	Long-term therapy with UFH reduces BMD and causes an increased fracture risk, while LMWH reduces BMD in the longer term but an increased fracture risk with LMWH has not been clearly demonstrated
Aromatase inhibitor	2. Reduce endogenous oestrogen leading to increased bone resorption	
Benzodiazepines and benzodiazepine-related drugs	1. Muscular weakness, sedation, extrapyramidal symptoms, imbalance and/or dizziness, visual disorders, delirium, orthostatic hypotension	
Centrally acting antihypertensives	1. Orthostatic hypotension, hypotension, syncope, dizziness/vertigo, sleep disorders, arrhythmia, weakness, drowsiness, sedation	
Diuretics	1. Orthostatic hypotension, hypotension, syncope, dizziness, sedation, electrolyte disturbances (hyponatremia), volume depletion	Increased calcium excretion not present in CKD
	2. Increased calcium excretion	
Glucocorticoids	1. Muscle wasting, visual impairment	Used for underlying kidney diseases and in transplantation
	2. Steroid induced osteoporosis	
	Decrease bone formation, increase resorption; impaired calcium/vitamin D metabolism	
Opioids	1. Orthostatic hypotension, hypotension, drowsiness, somnolence, dizziness or vertigo, sedation, confusion, delirium, eye disorders, muscle problems	Increased risk of opioid intoxication in CKD due to renal clearance
Overactive bladder and incontinence medications	1. Dizziness or vertigo, somnolence, delirium, visual impairment	
Proton pump inhibitors	1. Neuropathy due to low B12 absorption	Associated with increased risk of fracture in CKD
	2. Reduced gastrointestinal absorption of magnesium and calcium	
Thyroxine	2. Increased bone remodelling	
Vasodilators used in cardiac diseases	1. Hypotension, orthostatic hypotension, dizziness, rhythm disorders, somnolence and syncope	

BMD, bone mineral density; UFH, unfractionated heparin; LMWH, low‐molecular‐weight heparin.

Several classes of medications are associated with increased risk of falls (Table 2). These Fall-Risk-Increasing Drugs (FRIDs) includes psychotropics, antiepileptics, anticholinergics and some classes of cardiovascular medications [75]. Many of these are frequently used for patients with CKD. An important consideration in the setting of CKD is clearance by the kidneys, with risk of accumulation of the drugs and their metabolites if proper dose adjustments are not considered, which may further increase the risk of falls.

Systematic medication review and deprescribing should be prioritized in patients with CKD, especially in older and frail individuals [76], where such approaches have been shown to reduce the pill burden significantly [77]. Tools such as STOPPFall (Screening Tool of Older Persons Prescriptions in older adults with high fall risk) [78] may be helpful to identify FRIDs and guide the deprescribing process. When prescribing these drugs, falls risk should be carefully weighed against the expected benefits of the drug. Regular reassessment of the medication list for drugs associated with falls or known adverse effects in bone may further help reduce the risk of falls and fractures [75].

### Tip 7: Reduce home hazards and prevent falls

Non-pharmacological measures aiming to reduce home hazards, are of the utmost importance, as around half of all falls occur at home [79]. Those measures include, but are not limited to, proper lightening at home, avoiding slippery materials overall and particularly in bathrooms, removal of doorsteps and loose carpets, wearing appropriate footwear, as well as measures to ease ambulation and physical exercise. Home modifications have been found to reduce the number of falls by 7% [80]. In many countries, social services may perform home visits and do undertakings to prevent falls.

An aspect of specific importance for patients with CKD is the potential advantage of home-based therapy. Intradialytic hypotension has been established as a major risk factor for falls in patients receiving dialysis therapy [81, 82]. Home-based haemodialysis or peritoneal dialysis improve haemodynamic stability, potentially reducing post-dialysis hypotension and fatigue; increase flexibility of dialysis scheduling hypothetically improving physical activity and sleep quality; reduce regular travel needs and centre-related potential hazards; and are associated with less frailty as compared with in-centre treatment [83]. These findings may be due to selection bias and possible benefits must be balanced against the theoretical risk of increased falls risk in the home due to tubes, line, and other equipment necessary for home-based dialysis.

Eye and kidney diseases share multiple risk factors such as diabetes mellitus, hypertension and hyperlipidaemia [84]. Certain eye disorders are highly prevalent, including cataract in 33%–80% and age-related macular degeneration in 8%–36%, and any kind of vision impairment in 36%–60% of patients with CKD [85]. Vision impairment is linked to higher risk of falls and fractures [86]. Glasses for visual correction are recommended, although multifocal glasses are associated with increased risk of falls due to impaired depth perception and edge-contrast sensitivity [87].

The beneficial effects of cataract surgery on fall risk in the general population are well-established along with an additional benefit from second-eye surgery [88–90] providing a 16% reduction in hip fracture risk [91]. There is currently no prospective clinical study evaluating such benefit in patients with CKD. Nonetheless, vulnerability and higher fall rates in patients with CKD may provide even better outcomes for cataract surgery in this population.

### Tip 8: Explore for neuropathy

Peripheral neuropathy is common in CKD, with approximately 60%–90% of patients receiving dialysis being affected by peripheral neuropathy [92]. Sensory, motor and autonomic neuropathy have been described. Sensory nerve function may be impaired before motor nerve function [93], resulting in malfunctioning sensation and balance. Later, motor neuron function can also be impaired, contributing to muscle weakness, sarcopaenia and wasting [94]. Autonomic neuropathy can cause hypotension and cardiac arrhythmia, contributing to orthostatic instability.

Potential contributors to peripheral neuropathy in CKD include uraemic toxins, hyperkalaemia, and disturbed mineral metabolism with hyperparathyroidism. Reducing uraemic toxins by low-protein diet or dialysis treatment has the potential to improve or stabilize peripheral neuropathy [95, 96]. Dietary potassium restriction as well as parathyroidectomy have also been associated with improved peripheral nerve function [97, 98]. Vitamin B12 deficiency should be identified and treated if present.

Diabetes is among the most common causes of CKD. Both type 1 and type 2 diabetes are associated with increased fracture risk. In addition to a direct effect on bone quality, diabetes can damage nerve function, causing a peripheral neuropathy with a predominant sensory component, resulting in gait instability and increased risk of falls [99]. Improved glycaemic control can reduce the incidence and progression of diabetic neuropathy [100].

A patient with neuropathy should be advised to prevent falls especially according to ‘Tip 7: Prevent home hazards’ and ‘Tip 9: Awareness and management of orthostatic hypotension’.

### Tip 9: Awareness and management of orthostatic hypotension

Orthostatic hypotension is associated with an increased risk of falls and fractures [101] and is common in patients with CKD. In the CRIC (Chronic Renal Insufficiency Cohort) cohort, 5% of patients with CKD G2–4 (estimated glomerular filtration rate 20–70 mL/min/1.73 m^2^) had orthostatic hypotension [102]. Even higher prevalences have been reported in patients receiving dialysis, including 18% in those receiving peritoneal dialysis [103] and up to 42% in those receiving haemodialysis [104].

Patients with CKD have an increased burden of risk factors associated with orthostatic hypotension including older age, use of medications increasing the risk of orthostatic hypotension (Table 2) and autonomic failure [105]. In addition, the large haemodynamic changes due to fluid removal and reduction of the intravascular volume during a haemodialysis session increase the tendency to orthostatism.

Patients should be educated on preventive measures to reduce the risk of orthostatic hypotension including gradual transitions to standing and activation of the lower extremity musculature through physical manoeuvers [106]. Medication regimens should be systematically reviewed with deprescribing of harmful drugs where appropriate. Withholding antihypertensives on dialysis days should also be considered. In addition, large-volume fluid removal during a single haemodialysis session should be avoided whenever possible, for instance through fluid restrictions or by increasing dialysis duration or frequency. In the general population, rapid ingestion of water (approximately 500 mL) prior to triggering events may transiently increase blood pressure and thereby reduce symptoms of orthostatic hypotension [106]. This advice should be adjusted according to the residual kidney function in the individual patient with CKD.

The use of an α-1 adrenergic receptor agonist has been suggested to prevent intradialytic hypotension and orthostatic hypotension in patients on haemodialysis. Although a protective effect has been demonstrated, these studies were small and mostly not randomized [107], and observational data cautions a possible increased risk of cardiovascular events in treated patients [108].

### Tip 10: Explore for fall risk and implement fracture liaison services

Risk of fall should be explored in patients with CKD-associated osteoporosis (Fig. 3). The patient should be asked about falls during the last year, as a previous fall is associated with a high risk of recurrent fall. We recommend adhering to the World Falls Guideline especially for older people [109]. Risk assessment tools have been developed for patients on dialysis, but need further validation in other populations [110].

**Figure 3:**
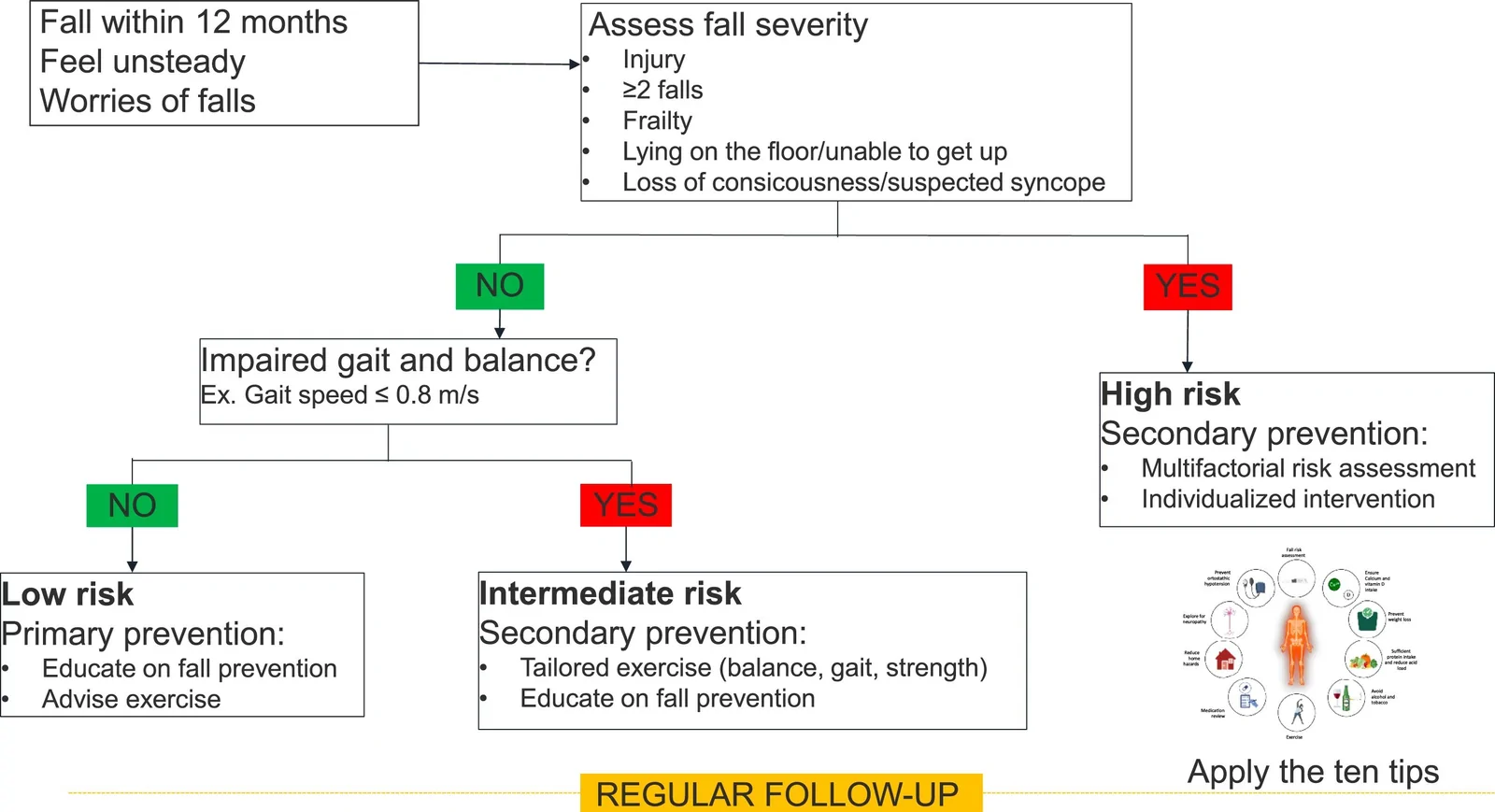
Fall risk assessment and intervention.

If patients are at increased risk of falls, gait and balance should be examined, so that a tailored exercise for balance, gait and strength could be provided if relevant. Underlying causes should be explored and, if possible, treated. Finally, fall preventive measures should be initiated [109]. A multidisciplinary approach will often be of relevance as multifactorial interventions including geriatric assessments and exercise are associated with a reduced risk of falls and fracture [111].

A multifactorial assessment should be performed to determine underlying risk factors for falls, and relevant interventions should be initiated. A modified algorithm based on [109] is suggested in (Fig. 3).

A Fracture Liaison Service (FLS) is a multidisciplinary, system-level framework designed to mitigate the risk of secondary fractures in patients who have recently sustained a fragility fracture. This proactive approach is critical because the risk of a subsequent fracture is highly time-dependent, peaking within the first 2 years after the initial injury [112].

The effectiveness of the FLS model is supported by significant meta-analytical data (Supplementary data, Table S1). Recent systematic reviews underscore the impact of FLS on patient outcomes. Danazumi *et al*. [113] reported moderate-certainty evidence indicating a significantly lower risk of secondary fragility fractures in FLS interventions compared with standard care at >2 years [risk ratio 0.68, 95% confidence interval (CI) 0.55–0.83]. Similarly, Li *et al*. [114] demonstrated that FLS care is associated with a lower probability of subsequent fractures [odds ratio (OR) 0.70, 95% CI 0.52–0.93], with the most pronounced benefits observed in studies with a follow-up exceeding 2 years (OR 0.57, 95% CI 0.34–0.94). Furthermore, their analysis identified a significant reduction in mortality within pre–post implementation comparisons (OR 0.65, 95% CI 0.44–0.95).

FLS programs systematically identify patients, assess their future fracture and falls risk, and ensure timely osteoporosis management. These services streamline access to bone health evaluations and the initiation of appropriate pharmacological treatment.

Despite the proven success of FLS in the general population, a critical gap remains regarding patients with CKD. Given the high prevalence of osteoporosis in CKDG4–5D and the frequent underdiagnosis of vertebral fractures in this cohort, the current FLS model requires evolution. In addition, CKD associates with worse patient-reported experience measures in patients who experience a fragility fracture [115]. To address these unique bone health challenges and ensure optimal care, inclusion of nephrological expertise in the FLS multidisciplinary team may be beneficial. Such an expansion of the network would facilitate specialized assessment and tailored treatment strategies for this complex patient population.

## Supplementary Material

sfag303_Supplemental_Files

## Data Availability

No new data were generated or analysed in support of this research
