# Supplementary material for: Ten tips to reduce fracture risk by non-pharmacologic interventions in CKD-associated osteoporosis
Source: Clin Kidney J. 2026 Sep 1;19(10):sfag303. doi: 10.1093/ckj/sfag303 (PMC13627839; doi:10.1093/ckj/sfag303)
Supplement: Supplementary file 1 [file sfag303_supplemental_files.zip › Summary slides.pdf]

Ten tips to reduce fracture risk by non-pharmacologic interventions in CKD-associated osteoporosis

Ditte Hansen[1,2], Hanne Skou-Jørgensen[3-5], Mehmet Kanbay[6], Maria Fusaro[7,8], Louise Salomo[1], Susanne Fleig[9], Stig Molsted[2,10], Jesper Ryg[2,11,12], Mathias Haarhaus[13,14], Pieter Evenepoel[15,16], on behalf of EUROD and the CKD-MBD working group of European Renal Association

1.Department of Nephrology, Copenhagen University Hospital - Herlev, Copenhagen, Denmark 2.Institute of Clinical Medicine, University of Copenhagen, Copenhagen, Denmark 3. Department of Clinical Medicine, Aarhus University, Aarhus, Denmark 4. Department of Renal Medicine, Aarhus University Hospital, Aarhus, Denmark 5. Department of Clinical Medicine, Aalborg University, Aalborg, Denmark 6. Department of Internal Medicine, Division of Nephrology, Koc University School of Medicine, Istanbul, Turky 7. National Research Council (CNR), Institute of Clinical Physiology (IFC), 56124 Pisa, Italy 8. Department of Medicine, University of Padova, 35128 Padova, Italy 9. Department of Nephrology and Clinical Immunology, RWTH Aachen University Hospital, Aachen, Germany 10. Department of Physiotherapy and Occupational Therapy, Copenhagen University Hospital – North Zealand, Denmark 11. Geriatric Research and Clinical Evidence (GRACE), Department of Internal Medicine Geriatric Section, Copenhagen University Hospital - Herlev and Gentofte Hospital, Copenhagen, Denmark 12. Geriatric Research Unit, Department of Clinical Research, University of Southern Denmark, Odense, Denmark 13. Division of Renal Medicine, Department of Clinical Science, Intervention and Technology, Karolinska Institutet, Karolinska Universitetssjukhuset, Stockholm, Sweden 14. Tiohundra AB, Norrtälje, Sweden 15 Department of Microbiology, Immunology and Transplantation, Nephrology and Renal Transplantation Research Group, Katholieke Universiteit Leuven, Leuven, Belgium 16 Department of Medicine, Division of Nephrology, University Hospitals Leuven, Leuven, Belgium

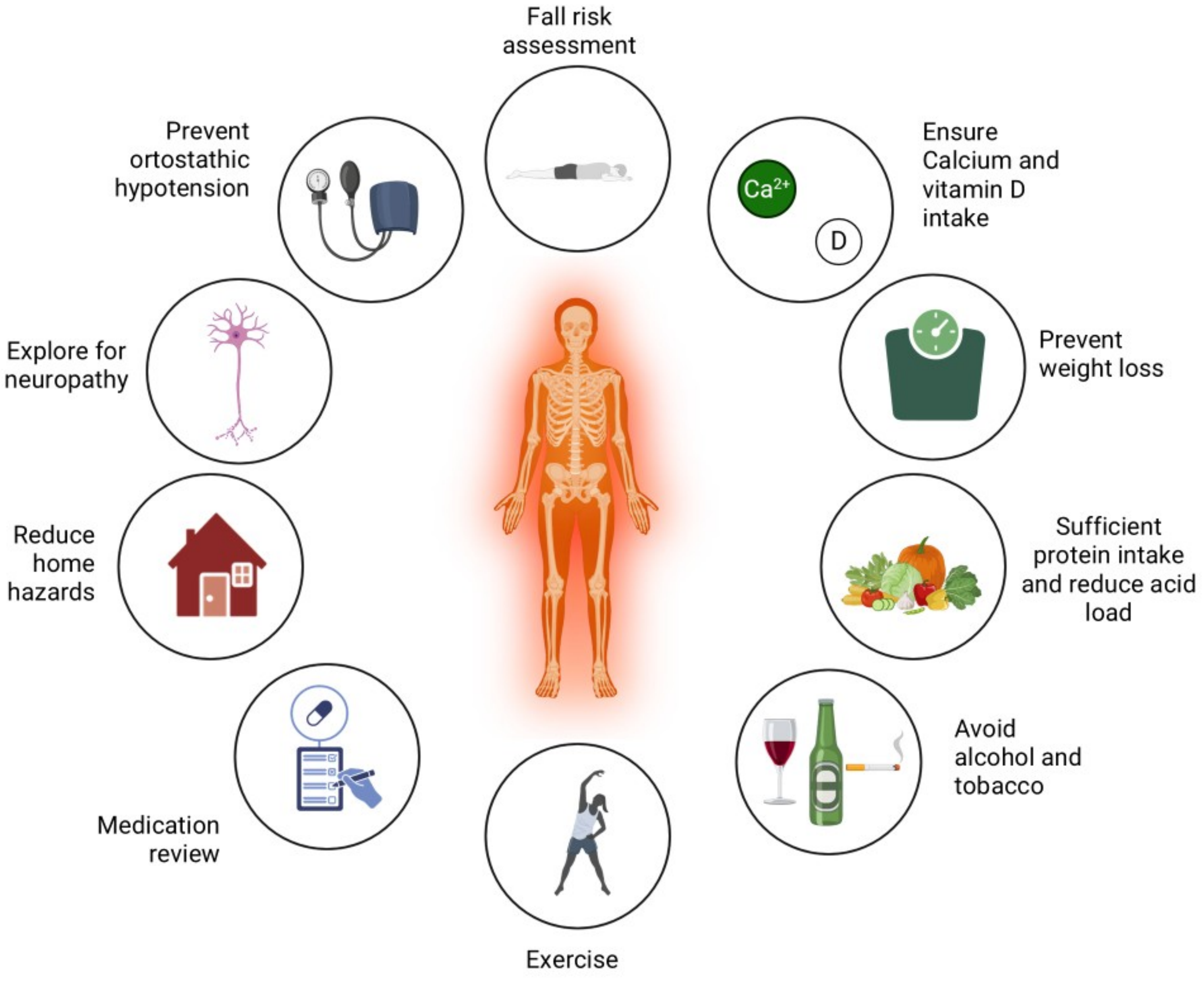

Figures created with the use of BioRender

TIP 1:
Ensure sufficient calcium and vitamin D intake

- A sufficient calcium intake is important for bone mineralisation
- The dietary calcium intake in adults with CKD G4-5D are often too low

----

- Vitamin D is important for
  - Calium uptake from the gut
  - Regulation of parathyroid hormone
  - Normal mineralisation of bone
- Nutritional vitamin D are often necessary to obtain adequate levels of 25-hydroxy vitamin D

Sufficient calcium intake

800-1000 mg/day

Calculate and supplement

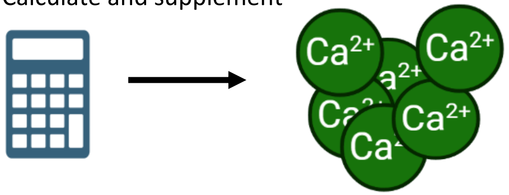

Sufficient vitamin D intake

Target 25-hydroxy vitamin D >75 nmol/L

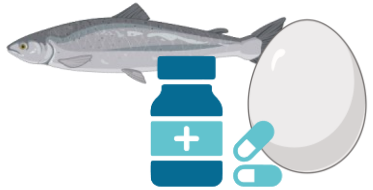

Evenepoel et al Nephrol Dial Transpl 2024; 39:341-366; Jørgensen et al Nephrolo Dial Transpl 2025; 40:797-822

TIP 2:
Ensure sufficient nutrition to prevent weight loss

- Weight loss is associated with increased risk of falls and fractures
- Inadequate energy intake accelerate muscle loss and skeletal fragility
- Monitor weight and BMI regularly in CKD G4-5D
- Provide individualized nutritional advice, preferable by renal dietitian.

Sufficient nutrition
Avoid weight loss

Warning!
>5% over 3–6 months

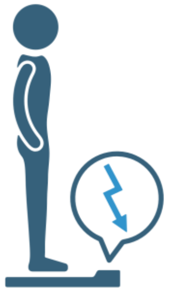

Koppe et al JCSM 2019; 10:479-757; Ikzler et al Am J Kidney Dis 2020; 76:s1-s107

TIP 3:
Ensure low acid load and sufficient protein intake

- Acidosis may cause bone mineral loss and accelerates muscle wasting
- Acidosis associates with increased risk of fracture in CKD
- Plant based food intake reduce acid load

- A sufficient protein intake preserves the muscle mass
- Consider to increase the protein intake in CKD G5D and in patients with sarcopenia

Preferable plant-based food

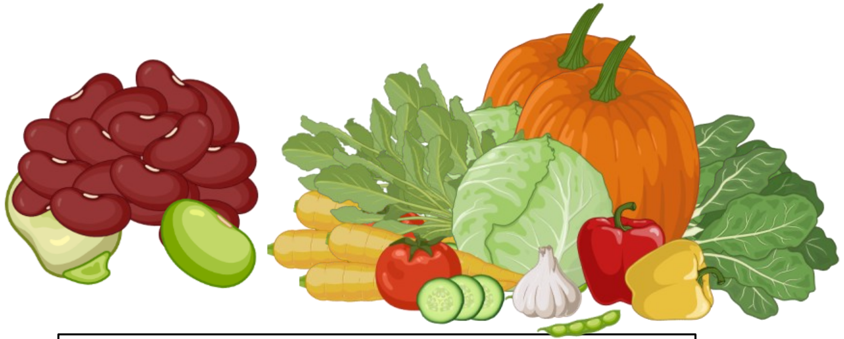

Reduce acid/uremic toxin load

Low phosphate/protein ratio

Increase gastrointestinal motility

Increase vitamin K intake

Sufficient protein intake

- CKD: ~ 0.8 g/kg*day
- Dialysis: ~ 1-1.2 g/kg*day
- Frail/sarcopenia: ~ 1-1.2 g/kg*day

Marhtur et la Clin Kidney J 2022;15: 1379-1386; Hansen NM et alJ Renal Nutrition 2023; 33:412-419; Steven et al Kidney Int 2024; 105:S117-S314

TIP 4:
Reduce smoking and alcohol

- Smoking is a risk factor for osteoporosis and fracture
- Smoking should be avoided

- Excessive alcohol intake impairs calcium absorption, increase risk of falls, cause hormonal disturbances
- Excessive alcohol intake should be discouraged to prevent fracture

Thorin et al Osteoporosis Int 2016;27:249-255; LeBoff et al Osteporosis Int 2022; 33:2049-2102

TIP 5:
Advice exercise training

- Exercise reduces falls and fracture in the general population
- Exercise associates with improved physical function and bone mineral density in patients with CKD
- Exercise is beneficial for over-all health

- Exercise should be recommended to patients with CKD and organized exercise programs available

Sherrington et al Cochrane Syst Rev 2019; Cardoso et al BMC Nephrology 2000; 21

TIP 6:
Conduct regular medication review and deprescribing

- Polypharmacy is associated with an increased risk of falls and fracture in CKD
- Some medications frequently used in CKD may reduce bone health, increase risk of falls and fracture
- Systematic medication review and deprescribing should be done in older and frail patients
- Tools such as STOPFALL are helpful to identify FRIDs (Fall Risk Increasing Drugs) and guide deprescribing

Van der Velde et al Eur Geriatric Med 2023:14; 649; Azzolino et al Jornal of Frailty and Aging 2023: 12:224-246; Seppala et al Age Aging 2021; 50:1189-1199

TIP 7:
Reduce home hazards and prevent falls

Provide social service to perform home visits and do undertakings for prevention of falls.

Consider fall risk when installing home dialysis equipment

Take care of:

- Proper lightning
- Avoid slippery materials on the floor
- Remove doorsteps and loose carpets
- Wear appropriate footwear
- Correct vision impairment

Clemson et al Cochrane Databse Syst Rev 2023; Lektip et al PeerJ 2023: 11

TIP 8:
Explore for neuropathy

Peripheral neuropathy is common in CKD

Neuropathy may increase the risk of falls

- Sensory neuropathy reduce sensation and balance
- Motor neuropathy may lead to muscle weakness
- Autonomic neuropathy can cause hypotension

Identify neuropathy and take cautions to prevent falls

Krishnan et al Nat Rev Neurol 2019:34:625-632

TIP 9:
Awareness and management of orthostatic hypotension

Orthostatic hypotension increases risk of fall and fracture

Educate patients with orthostatic hypotension:

- Gradual transitions
- Activate lower extremity musculature when raising
- Deprescribe harmful drugs if possible
- Fluid restriction and increased dialysis duration if large fluid removals are required during dialysis
- Avoid antihypertensives on dialysis days

Juraschek JAMA Int Med 2017;177:1316-1323; Wieling et la Lancet Neurol 2022; 21:735-746

TIP 10:
Explore for fall risk

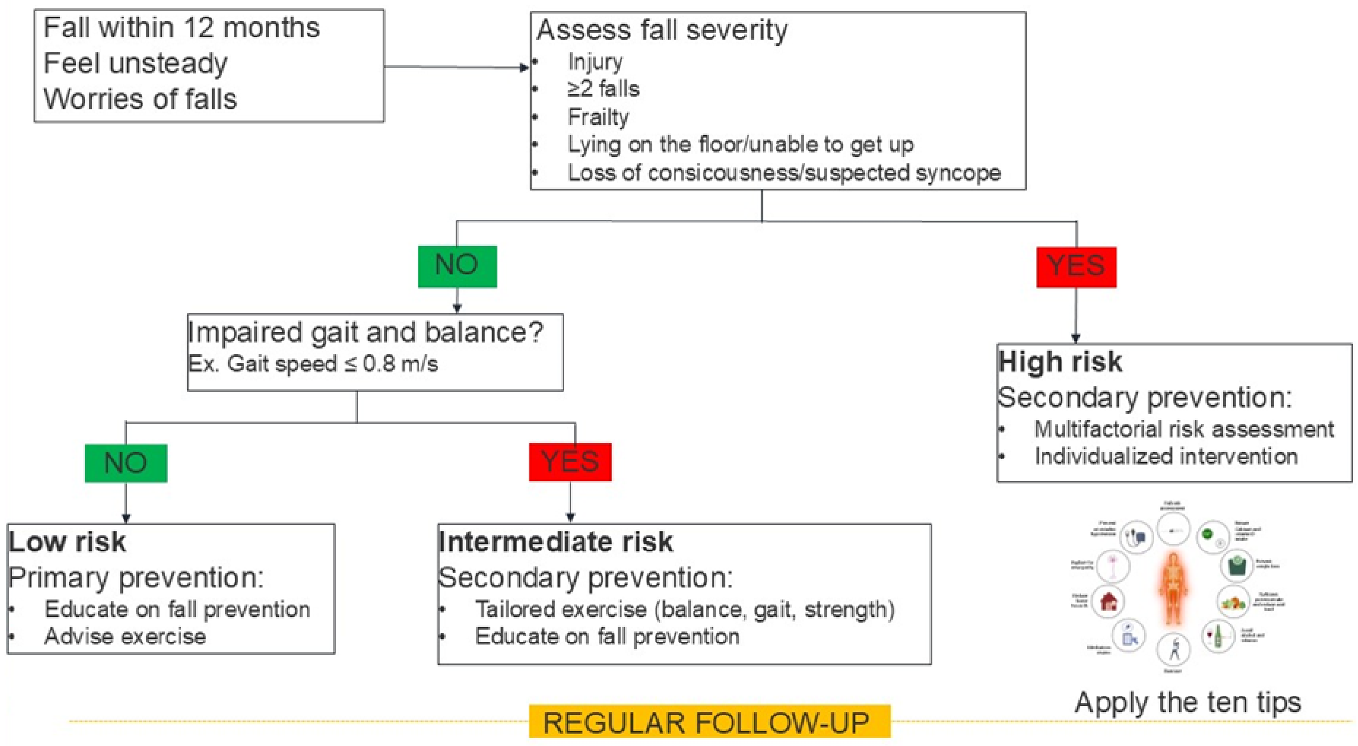

Modified from Montero-Odasso Age Aging 2022; 51

TIP 10:
and Implement fracture liaison services

Fracture Liaison Service (FLS) is

A multidisciplinary, system-level framework designed to mitigate the risk of secondary fractures in patients who have recently sustained a fragility fracture

FLS reduces risk of secondary fracture

FLS should be avalible for patients with CKD

Nephrological expertise should be included in the FLS team

Danazumi et al Osteoporosis Int 2024; 35:1133-1151
