# Supplementary material for: Ten tips to reduce fracture risk by non-pharmacologic interventions in CKD-associated osteoporosis
Source: Clin Kidney J. 2026 Sep 1;19(10):sfag303. doi: 10.1093/ckj/sfag303 (PMC13627839; doi:10.1093/ckj/sfag303)
Supplement: Supplementary file 1 [file sfag303_supplemental_files.zip › Supplementary table 1.docx]

Supplemental material Table 1s

**Summary of Evidence and Strategic Recommendations for FLS in CKD patients**

| **Feature** | **Clinical Evidence & Implementation** | **Source / Rationale** |
| --- | --- | --- |
| **Core Objective** | Reduction of secondary fragility fractures through a multidisciplinary, system-level approach. | General FLS Framework |
| **Critical Window** | Highest risk of second fracture occurs within the first 2 years post-initial event. | Clinical Guidelines |
| **Long-term Efficacy** | RR 0.68 (95% CI 0.55–0.83) for secondary fractures at > 2 years; moderate certainty evidence. | Danazumi et al. (2024)[108] |
| **Fracture Reduction** | OR 0.70 (95% CI 0.52-0.93) (overall) and OR 0.57 (95% CI 0.34-.94) (for follow-up > 2 years) in post-FLS implementation. | Li et al. (2021)[109] |
| **Mortality Benefit** | OR 0.65 (95% CI 0.44-0.94) significantly lower probability of mortality in pre-post FLS comparisons. | Li et al. (2021)[109] |
| **The CKD Gap** | High prevalence of osteoporosis and severe underdiagnosis of vertebral fractures (VFs). | Clinical Observation |
| **Network Expansion** | Mandatory inclusion of a Nephrologist in the multidisciplinary team for advanced CKD. | Strategic Recommendation  Fusaro et al (2025)[107] |
